# Impact of left ventricular ejection fraction on unplanned readmission after aortic regurgitation surgery: a single-center retrospective cohort study

**DOI:** 10.3389/fcvm.2026.1714384

**Published:** 2026-04-21

**Authors:** Pan Li, Yong-jian Zhang, Feng-wei Guo, Hong Chen, Ya-ling Dong, Jia-nan Shang, Hui-rong Wu, Fei Wang, Meng Chen, Li-tao Ruan, Yang Yan, Yan Song

**Affiliations:** 1 Department of Ultrasound, The First Affiliated Hospital of Xi’an Jiaotong University, Xi’an, Shaanxi, China; 2 Department of Cardiovascular Surgery, The First Affiliated Hospital of Xi’an Jiaotong University, Xi’an, Shaanxi, China

**Keywords:** aortic regurgitation, left ventricular dilation, left ventricular ejection fraction, mild impairment, unplanned readmission

## Abstract

**Background and objective:**

Aortic regurgitation (AR) is a common valvular heart disease. Despite advances in surgical techniques, unplanned readmission rates after surgery remain high. This study aimed to investigate the risk of postoperative unplanned readmission in patients with only mildly impaired left ventricular ejection fraction (LVEF 55%–60%) but concomitant left ventricular enlargement.

**Methods:**

This single-center retrospective cohort study enrolled 841 adult patients diagnosed with aortic regurgitation who underwent surgical treatment at our hospital between January 2020 and December 2024. All patients underwent transthoracic echocardiography (TTE) within 3 days before surgery. The primary endpoint event was unplanned readmission during postoperative follow-up.

**Results:**

During follow-up, 46 patients experienced unplanned readmission. Risk factor analysis for readmission indicated that patients in the LVEF 55%–60% group had significantly higher risk of postoperative readmission [HR (95% CI): 4.118 (1.488–11.397), *P* = 0.006]. Further Cox proportional hazards regression analysis revealed that LVEDD > 65 mm and LVEF <60% were significant risk factors for unplanned readmission during postoperative follow-up (*P* < 0.05). Among all patients with unplanned readmissions, 21 cases had both LVEDD > 65 mm and LVEF < 60%, of whom 12 patients (57.14%) had an LVEF of 55%–60%. Cox regression analysis showed that patients meeting both risk factors had a significantly higher risk of unplanned readmission compared to those with neither risk factor [HR (95% CI): 2.548 (1.174–5.534), *P* = 0.018].

**Conclusion:**

Patients with mildly impaired left ventricular ejection fraction (LVEF 55%–60%) combined with significant left ventricular dilation (LVEDD >65 mm) constitute a high-risk cohort for unplanned readmission postoperatively. For patients with aortic regurgitation, even those exhibiting only mildly impaired LVEF, coexisting significant left ventricular dilation warrants heightened vigilance regarding readmission risk.

## 1 Background

Aortic regurgitation (AR) is a common valvular heart disease. A 20-year follow-up study showed a cumulative incidence of 14.5% for moderate or severe aortic regurgitation (1). AR patients often develop left ventricular (LV) enlargement due to volume overload, while their left ventricular ejection fraction (LVEF) remains mildly impaired or near-normal. Current guidelines recommend surgical intervention when patients develop symptoms, significant LVEF reduction (<55%), or severe LV dilation [end-diastolic diameter [LVEDD] > 65 mm or end-systolic diameter [LVESD] > 50 mm] (2, 3). However, clinical management remains controversial for patients with mildly impaired LVEF (55%–60%) who already present left ventricular enlargement (2). The long-term prognosis, particularly postoperative outcomes, in this subgroup has not been fully elucidated (4). Despite undergoing aortic valve surgery, these patients' postoperative risks—especially unplanned readmission rates—may be overlooked. Left ventricular enlargement indicates underlying myocardial remodeling and functional abnormalities, which may impair postoperative recovery and reverse remodeling even with preserved LVEF. This increases risks of heart failure, arrhythmia, and other complications leading to readmission (5–7). Therefore, comprehensively investigating postoperative readmission risks and influencing factors holds significant clinical importance for optimizing surgical timing and improving long-term prognosis. This study aims to evaluate unplanned readmission risk after surgery in patients with left ventricular enlargement despite mildly impaired LVEF (55%–60%).

## 2 Materials and methods

### 2.1 Study population

This single-center retrospective cohort study enrolled adult patients diagnosed with aortic regurgitation who underwent surgical aortic valve replacement or transcatheter aortic valve replacement at our institution between January 2020 and December 2024.

Inclusion criteria: (1) Diagnosed with moderate or severe aortic regurgitation in our cardiovascular surgery department; (2) Patients undergoing first-time aortic valve replacement or repair. Exclusion criteria: (1) Age < 18 years; (2) Complex congenital heart disease; (3) Previous aortic or mitral valve surgery or intervention; (4) Combined with moderate or above aortic stenosis; (5) Pre-existing severe primary stenosis or regurgitation of other valves; (6) Patients with infective endocarditis. The study protocol was approved by the Ethics Committee of the First Affiliated Hospital of Xi'an Jiaotong University.

### 2.2 Echocardiographic assessment

All patients underwent transthoracic echocardiography within 3 days before surgery. Examinations were performed using a Philips CX50/7C color Doppler ultrasound system equipped with S5-1 or X5-1 transducers. Aortic regurgitation severity was classified according to guideline recommendations (8). Additional evaluated parameters included: Left ventricular structure and function: left ventricular end-diastolic diameter (LVEDD) and volume (LVEDV), left ventricular end-systolic diameter (LVESD) and volume (LVESV), left ventricular ejection fraction (LVEF); Atrial size: left atrial and right atrial dimensions; Right ventricular function: Tricuspid annular plane systolic excursion (TAPSE), Pulmonary artery systolic pressure (PASP). Left ventricular ejection fraction was measured using the biplane Simpson's method. For patients with atrial fibrillation, the average value was calculated over 3–5 consecutive cardiac cycles to account for beat-to-beat variability. All measurements were performed in accordance with the guidelines of the European Association of Cardiovascular Imaging and the American Society of Echocardiography (9).

To assess inter-observer variability, a random sample of 50 patients was independently re-measured by a second experienced echocardiographer blinded to clinical data. The intraclass correlation coefficient (ICC) for LVEF was 0.92 (95% CI: 0.87–0.96), and for LVEDD was 0.90 (95% CI: 0.84–0.92), indicating excellent reproducibility.

### 2.3 Clinical data collection and endpoints

The following data were collected through the electronic medical record system: Baseline characteristics: demographic features (age, sex, height, weight), blood pressure, comorbidities (hypertension, diabetes, chronic kidney disease, cardiovascular history), preoperative laboratory parameters (including creatinine (Cr), blood urea nitrogen (BUN), estimated glomerular filtration rate (eGFR), N-terminal pro-brain natriuretic peptide (NT-proBNP)), electrocardiogram findings, and surgical approaches.

Primary endpoint event: Postoperative unplanned readmission. Defined as rehospitalization for any rehospitalization occurring >30 days after initial discharge for unplanned medical reasons(excluding routine follow-up visits, scheduled secondary surgeries, regular dialysis, etc.). Readmission information was extracted and confirmed via electronic medical record system follow-up data.

### 2.4 Statistical analysis

Statistical analysis was performed using SPSS 23.0 software. Categorical variables were described using frequencies and percentages. Since continuous variables showed non-normal distribution by Shapiro–Wilk test, they were expressed as median and interquartile range (IQR). To compare baseline characteristics across different left ventricular ejection fraction (LVEF) strata, continuous variables were analyzed using the Kruskal–Wallis H test (non-parametric analysis of variance), while categorical variables were assessed using the Chi-square test (*χ*² test). Logistic regression analysis was used to evaluate risk factors for unplanned readmission following AR surgery. Cox proportional hazards regression analysis further validated the predictive efficacy of risk factors for readmission, with Kaplan–Meier survival curves generated; All statistical analyses employed two-sided tests, with *P* < 0.05 indicating statistical significance.

## 3 Results

### 3.1 Analysis of baseline characteristics by LVEF values

**Figure 1:**
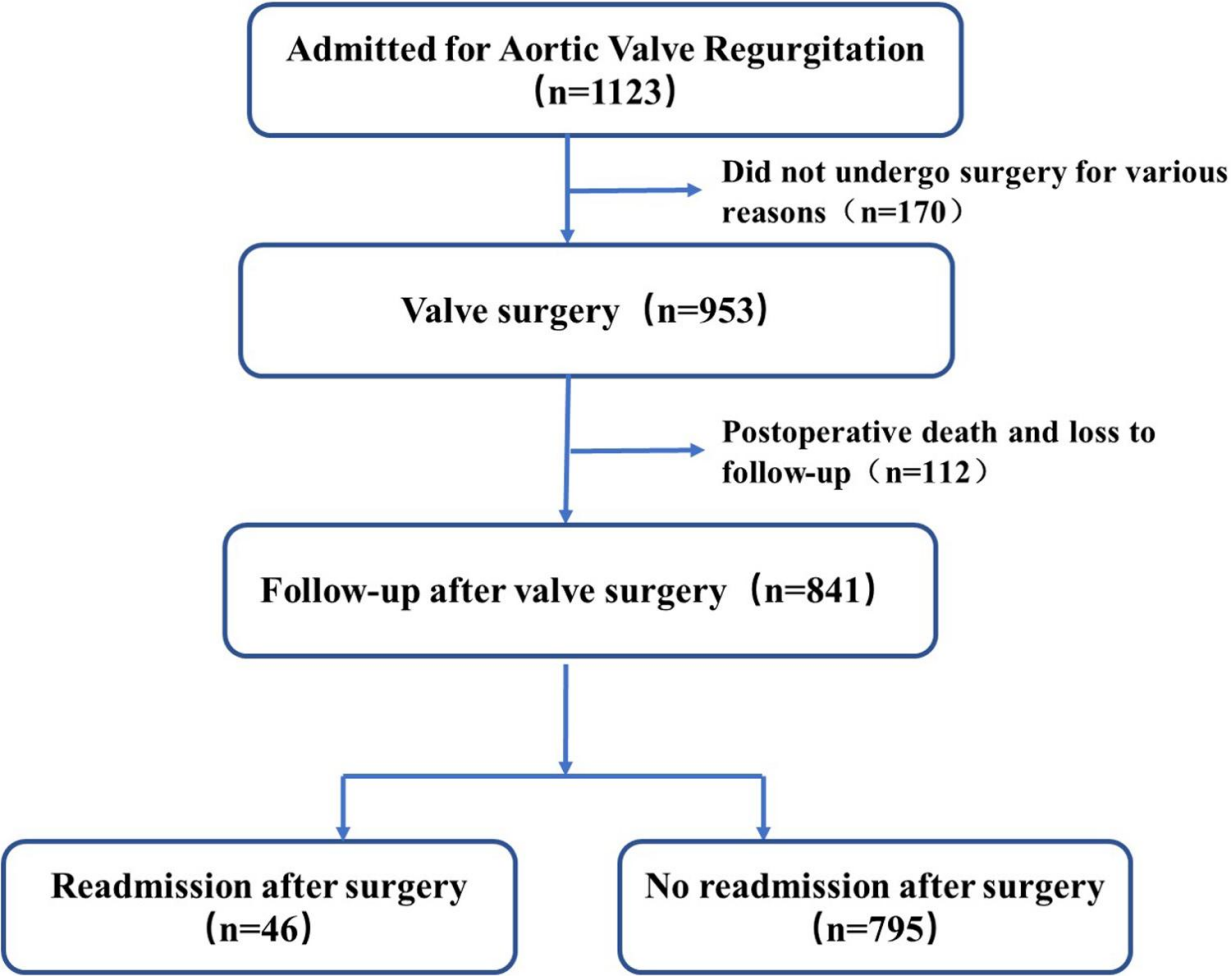
Research flowchart.

This retrospective study analyzed 1,123 adult patients diagnosed with aortic regurgitation at our center. After excluding 170 patients with mild regurgitation or non-surgical management, and 112 patients with postoperative mortality or lost to follow-up, a total of 841 patients were ultimately enrolled. The maximum follow-up duration was 58 months. During follow-up, 46 patients experienced unplanned readmission. The detailed research flowchart is shown in Figure 1.

In the unplanned readmission analysis cohort, 37 patients (80.4%) were male, with an age range of 27–81 years and a mean age of 56 years. 31 patients (67.39%) required readmission within one year after discharge, 7 patients (15.22%) experienced unplanned readmission within the second year, and 8 patients (17.39%) had unplanned readmission >3 years postoperatively. The median follow-up duration was 9 months (IQR: 4–16 months), with a maximum follow-up of 48 months. Among the 46 readmitted patients, the specific causes were: heart failure (*n* = 18, 39.1%), postoperative fever/infection (*n* = 12, 26.1%), arrhythmias (*n* = 8, 17.4%), bioprosthetic valve vegetation (*n* = 4, 8.7%), mechanical valve thrombosis (*n* = 2, 4.3%), and other causes (*n* = 2, 4.3%). Notably, cardiac-related causes (heart failure + arrhythmias) accounted for 56.5% of readmissions, supporting the association between LV dysfunction and readmission risk.

Table 1 presents the baseline characteristics of the study population stratified by left ventricular ejection fraction (LVEF). The study population was stratified into subgroups based on LVEF levels: LVEF ≥ 70% (*n* = 87), 60%–70% (*n* = 355), 55%–60% (*n* = 150), 50%–55% (*n* = 93), and <50% (*n* = 156). Intergroup comparisons revealed that both systolic and diastolic blood pressure showed a progressive increase with higher LVEF levels, with statistically significant differences among groups (*P* < 0.001). The preoperative atrial fibrillation prevalence was highest in the LVEF < 50% group, showing statistically significant intergroup differences (*P* < 0.001). Both BUN and Pro-BNP levels peaked in the LVEF < 50% group and progressively decreased with increasing LVEF values; The eGFR was lowest in the LVEF < 50% group and progressively increased with higher LVEF values, showing statistically significant differences between groups (*P* < 0.001). The types of prosthetic valves implanted showed no statistically significant difference between groups (*P* > 0.05).

**Table 1:** Baseline characteristics according to LVEF Strata M (IQR).

Characteristics	LVEF < 50%	LVEF 50%–55%	LVEF 55%–60%	LVEF 60%–70%	LVEF ≥ 70%	*P*
	(*n* = 156)	(*n* = 93)	(*n* = 150)	(*n* = 355)	(*n* = 87)	
Age, y	58 (51–65)	56.0 (48.50–64.0)	57.0 (49.0–64.0)	55.0 (46.0–64.0)	56.0 (48.0–62.0)	0.072
Sex males (male, %)	129 (82.70)	86 (92.50)	127 (84.70)	286 (80.60)	65 (74.70)	0.039
BMI (kg/m^2^)	23.23 (20.78–25.83)	24.16 (21.79–26.58)	23.76 (20.89–25.63)	23.88 (22.09–26.12)	23.88 (21.72–25.80)	0.147
Systolic pressure (mmHg)	126.0 (112.0–138.75)	134.0 (120.0–145.0)	134.0 (121.0–145.0)	133.0 (121.0–146.0)	131.(117.0–140.0)	<0.001
Diastolic pressure (mmHg)	64.0 (59.0–73.0)	66.0 (56.50–75.0)	67.50 (58.0–77.25)	71.0 (63.0–79.0)	73.0 (65.0–80.0)	<0.001
Hypertension (*n*, %)	71 (45.50)	58.0 (65.40)	94 (62.70)	188 (53.0)	52 (59.80)	0.189
Diabetes(*n*, %)	9 (5.80)	5 (5.40)	12 (8.0)	26 (7.30)	7 (8.0)	0.347
Coronary heart disease (*n*, %)	50 (33.10)	36 (40.40)	53 (37.10)	94 (27.60)	25 (30.5)	0.105
Atrial fibrillation (*n*, %)	24 (15.40)	7 (7.50)	15 (10.0)	14 (4.0)	7 (8.0)	<0.001
NYHA (IV, %)	142 (91.0)	80 (86.0)	127 (84.70)	287 (80.80)	72 (82.80)	0.006
Cr (μmol/L)	6.81 (5.52–8.45)	6.34 (5.48–7.57)	6.48 (5.09–7.86)	6.16 (5.19–7.23)	6.21 (5.14–7.31)	0.001
BUN (mmol/L)	73.0 (62.0–91.75)	72.0 (63.0–81.0)	67.50 (59.0–80.25)	65.0 (58.0–75.0)	66.50 (55.0–76.50)	<0.001
eGFR (mL/min/1.73 m²)	96.89 (78.10–104.78)	98.43 (89.11–107.59)	100.41 (88.48–107.56)	101.0 (93.60–110.53)	100.68 (90.01–111.59)	<0.001
Pro-BNP (ng/L)	2,000.0 (614.0–5,715.0)	1,045.0 (297.0–2,360.0)	405.0 (175.0–1,350.0)	200.50 (107.75–499.25)	206.0 (76.50–758.0)	<0.001
Aortic valve surgery						0.101
AVR (*n*, %)	155 (99.40)	92 (98.90)	147 (98.0)	350 (98.60)	83 (95.40)	
AVP (*n*, %)	1 (0.60)	1 (1.10)	3 (2.00)	5 (1.40)	4 (4.60)	
Prosthetic valve type						0.234
Bioprosthetic	111 (71.61)	58 (62.04)	102 (69.38)	226 (64.57)	55(66.26)	
Mechanical	44 (28.38)	34 (36.96)	45 (30.61)	124 (35.43)	28 (33.73)	

BMI, body mass index; NYHA, New York heart association; Cr, creatinine; BUN, blood urea nitrogen; eGFR, estimated glomerular filtration rate; Pro-BNP, B-type amino-terminal natriuretic peptide pro; Aortic valve replacement (AVR); AVP, aortic valve repairs.

### 3.2 Analysis of baseline echocardiographic parameters across different LVEF values

Baseline echocardiographic characteristics of the study population, stratified by LVEF, are presented in Table 2. Progressive left ventricular enlargement was observed with declining LVEF, as indicated by significant increases in ventricular dimensions (LVEDD, LVESD, iESD), volumes (LVEDV, LVESV, iESV), and left atrial size (anteroposterior and mediolateral diameters), along with the right atrial mediolateral diameter (*P* < 0.001). The prevalence of patients meeting the criteria for iESD ≥ 20 mm/m² and iESV ≥ 45 mL/m^2^ was also significantly higher in the lower LVEF cohorts (*P* < 0.001). Severe aortic regurgitation was more common in the LVEF 50%–55% group (88.2%) than in the LVEF < 50% group (87.0%). Despite marked left ventricular dysfunction, TAPSE values remained within the normal range for all groups, although significant differences between the groups were detected. Pulmonary artery systolic pressure was mildly elevated in both the LVEF < 50% and 50%–55% groups.

**Table 2:** Baseline echocardiographic characteristics according to LVEF Strata M (IQR).

Parameter	LVEF < 50%	LVEF 50%–55%	LVEF 55%–60%	LVEF 60%–70%	LVEF ≥ 70%	*P*
	(*n* = 156)	(*n* = 93)	(*n* = 150)	(*n* = 355)	(*n* = 87)	
LVEDD (mm)	72.0 (64.8–79.3)	68.0 (63.0–73.50)	63.0 (58.0–69.0)	58.0 (53.0–63.0)	56.0 (52.0–60.75)	<0.001
LVESD (mm)	56.0 (51.0–63.0)	49.50 (45.75–53.0)	43.0 (39.0–48.0)	37.0 (34.0–40.50)	32.50 (29.0–35.0)	<0.001
LVEDV (mL)	272.16 (214.13–337.46)	239.24 (201.19–285.09)	201.19 (166.56–247.26)	166.56 (135.34–201.19)	153.66 (129.51–185.19)	<0.001
LVESV (mL)	153.66 (123.81–201.19)	112.81 (91.89–135.34)	83.07 (65.91–107.52)	58.13 (47.43–72.11)	42.55 (32.21–50.87)	<0.001
LVEF (%)	38.0 (27–46)	53.0 (51.0–54.0)	57.0 (56.0–58.0)	64.0 (62.0–67.0)	72.0 (71.0–74.0)	<0.001
LVEDD > 65mm	110 (71.40)	55 (61.10)	64 (43.50)	48 (13.80)	11 (13.10)	<0.001
LVESD > 50 mm	121 (78.60)	41 (45.60)	19 (12.90)	6 (1.70)	1 (1.2)	<0.001
iESD (mm/m^2^)	32.82 (29.54–36.92)	27.55 (24.95–30.70)	25.21 (21.90–27.82)	21.28 (18.97–23.76)	18.38 (16.56–20.57)	<0.001
iESV (mL/m^2^)	89.70 (72.62–116.88)	65.48 (51.51–77.40)	48.59 (37.55–58.63)	33.77 (26.34–40.78)	23.48 (18.89–30.05)	<0.001
iESD ≥ 20 (mm/m^2^)	154 (98.70)	87 (97.80)	131 (87.30)	220 (63.0)	29 (34.50)	<0.001
iESV ≥ 45 (mL/m^2^)	150 (97.40)	76 (85.40)	87 (58.0)	65 (18.60)	2 (2.40)	<0.001
TAPSE	20.0 (17.0–24.0)	23.0 (21.0–26.0)	23.0 (19.0–25.0)	23.0 (20.0–25.0)	23.0 (19.0–26.0)	0.002
PASP (mmHg)	33.0 (26.0–42.25)	34.0 (25.0–41.0)	28.0 (23.0–35.0)	29.0 (21.0–34.0)	30.50 (24.0–35.0)	0.015
LA AP diameter (mm)	38.0 (35.0–43.0)	38.0 (34.50–42.0)	36.0 (32.0–39.0)	34.0 (30.0–37.0)	34.0 (30.0–37.0)	<0.001
LA LR diameter (mm)	45.0 (41.0–52.0)	44.0 (41.0–47.0)	42.0 (38.0–47.0)	40.0 (35.0–44.50)	40.0 (37.0–47.0)	<0.001
RA LR diameter (mm)	37.0 (34.0–41.0)	39.0 (35.0–43.0)	36.0 (33.0–39.0)	35.0 (31.0–38.0)	32.0 (30.0–40.0)	<0.001
AR (Severe, %)	136 (87.20)	82 (88.20)	117 (78.00)	220 (62.00)	44 (50.60)	<0.001

LVEDD, left ventricular end-diastolic diameter; LVESD, left ventricular end-systolic diameter; LVEDV, left ventricular end-diastolic volume; LVESV, left ventricular end-systolic volume; LVEF, left ventricular ejection fraction; LVEDD, left ventricular end-diastolic diameter; LVESD, left ventricular end-systolic diameter; iESD, indexed end-systolic diameter; iESV, indexed end-systolic volume; TAPSE, tricuspid annular plane systolic excursion; PASP, pulmonary artery systolic pressure; LA AP diameter, left atrial anteroposterior diameter; LA LR diameter, left atrial left-right diameter; RA LR diameter, right atrial left-right diameter; AR, aortic regurgitation.

### 3.3 Analysis of factors associated with unplanned readmission

Cox proportional hazards regression analysis for readmission demonstrated that patients in the LVEF 55%–60% group had a significantly higher risk of postoperative readmission than those in the reference group (LVEF < 50%) (HR = 4.118, *P* = 0.006) (Table 3). Subsequent analysis further identified LVEDD > 65 mm and LVEF <60% as risk factors for unplanned readmission during postoperative follow-up (*P* < 0.05). In contrast, patient sex, blood pressure, atrial fibrillation, renal function, atrial size, and right ventricular function were not significantly associated with the risk of unplanned readmission (*P* > 0.05) (Table 4).

**Table 3:** LVEF strata analysis to predict readmission status.

LVEF strata	B	Odds ratio (95%CI)	*P*
LVEF < 50%	1.00 (Ref.)	1.00 (Ref.)	0.001
LVEF 50%–55%	1.045	2.842 (0.901–8.963)	0.075
LVEF 55%–60%	1.415	4.118 (1.488–11.397)	0.006
LVEF 60%–70%	−0.133	0.875 (0.294–2.605)	0.811
LVEF ≥ 70%	0.611	1.841 (0.518–6.547)	0.345

LVEF, left ventricular ejection fraction.

**Table 4:** Univariate logistic regression analysis to predict readmission status.

Variable	B	HR (95% CI)	*P*
Sex males	0.138	1.148 (0.542–2.433)	0.719
Systolic pressure (mmHg)	0.002	1.002 (0.986–1.017)	0.840
Diastolic pressure (mmHg)	−0.002	0.998 (0.975–1.022)	0.864
Atrial fibrillation	−0.231	0.794 (0.240–2.630)	0.705
NYHA (IV,%)	0.048	1.049 (0.459–2.399)	0.909
Cr (μmol/L)	0.019	1.019 (0.937–1.108)	0.662
BUN (mmol/L)	0.002	1.002 (0.999–1.004)	0.204
eGFR (mL/min/1.73 m²)	−0.009	0.991 (0.979–1.004)	0.169
LVEDD (mm)	0.006	1.006 (0.978–1.035)	0.673
LVESD (mm)	−0.008	0.992 (0.963–1.022)	0.601
LVEDV (mL)	0.000	1.000 (0.996–1.003)	0.861
LVESV (mL)	−0.002	0.998 (0.991–1.004)	0.431
iESD (mm/m^2^)	0.002	1.002 (0.955–1.051)	0.935
iESV (mL/m^2^)	−0.002	0.998 (0.988–1.008)	0.683
LVEDD > 65 mm	0.846	2.331 (1.281–4.243)	0.006
LVESD > 50 mm	0.188	1.207 (0.612–2.381)	0.587
iESD ≥ 20 (mm/m^2^)	0.277	1.320 (0.624–2.789)	0.468
iESV ≥ 45 (mL/m^2^)	−0.169	0.844 (0.460–1.551)	0.585
LVEF < 60%	0.777	2.176 (1.167–4.055)	0.014
TAPSE	0.022	1.023 (0.923–1.133)	0.670
PASP (mmHg)	−0.017	0.983 (0.953–1.014)	0.277
LA AP diameter (mm)	−0.010	0.990 (0.943–1.041)	0.705
LA LR diameter (mm)	0.037	1.037 (0.994–1.083)	0.093
RA LR diameter (mm)	−0.023	0.977 (0.916–1.043)	0.493
AR (Severe, %)	−0.076	0.927 (0.510–1.683)	0.802

LVEDD, left ventricular end-diastolic diameter; LVESD, left ventricular end-systolic diameter; LVEDV, left ventricular end-diastolic volume; LVESV, left ventricular end-systolic volume; LVEF, left ventricular ejection fraction; LVEDD, left ventricular end-diastolic diameter; LVESD, left ventricular end-systolic diameter; iESD, indexed end-systolic diameter; iESV, indexed end-systolic volume; TAPSE, tricuspid annular plane systolic excursion; PASP, pulmonary artery systolic pressure; LA AP diameter, left atrial anteroposterior diameter; LA LR diameter, left atrial left-right diameter; RA LR diameter, right atrial left-right diameter; AR, aortic regurgitation.

### 3.4 Predictive value of risk factors for unplanned readmission

**Figure 2:**
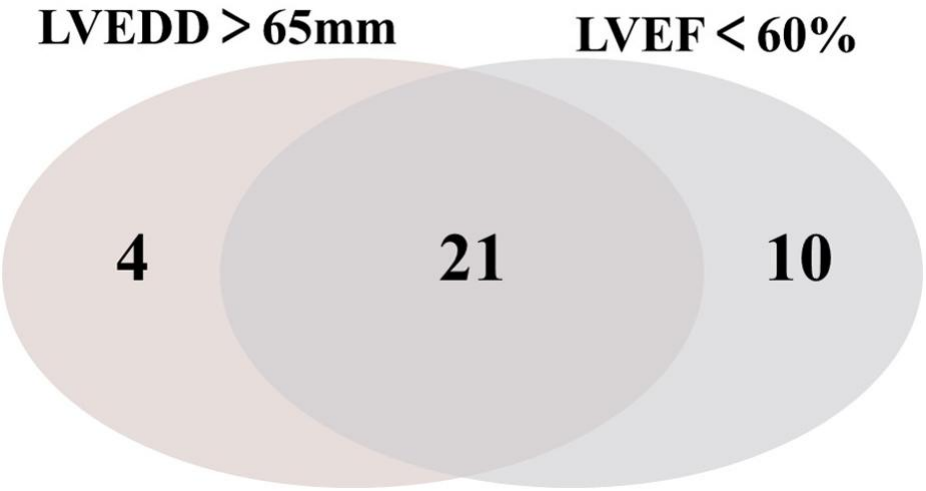
Prevalence of echocardiographic markers of unplanned readmission in patients after AR surgery Venn diagram showing the 2 markers of unplanned readmission: left ventricular end-diastolic diameter (LVEDD) > 65 mm, left ventricular ejection fraction (LVEF) < 60% and their overlap. AR, aortic regurgitation.

Figure 2 shows the distribution and overlap of LVEDD > 65 mm and LVEF < 60% among patients with unplanned readmissions during postoperative follow-up. Fourteen patients who met only one risk factor (either LVEDD > 65 mm or LVEF < 60%) were readmitted unexpectedly. Twenty-one patients who met both risk factors experienced unplanned readmissions. Cox proportional hazards regression analysis revealed that compared to patients without these risk factors, those meeting one risk factor showed increased risk of unplanned readmission after surgery, though without statistical significance [HR (95% CI): 1.231 (0.546–2.777), *P* = 0.616]. The risk of unplanned readmission was significantly elevated in patients meeting both risk factors, demonstrating a statistically significant difference [HR (95% CI): 2.548 (1.174–5.534), *P* = 0.018]. Detailed analysis is presented in Table 5.

**Table 5:** Association of markers analysis to predict readmission status.

Number of criteria met	B	HR (95% CI)	*P*
None meeting criteria	1.00 (Ref.)	1.00 (Ref.)	
1 meeting criteria	0.208	1.231 (0.546–2.777)	0.616
2 meeting criteria	0.935	2.548 (1.174–5.534)	0.018

**Figure 3:**
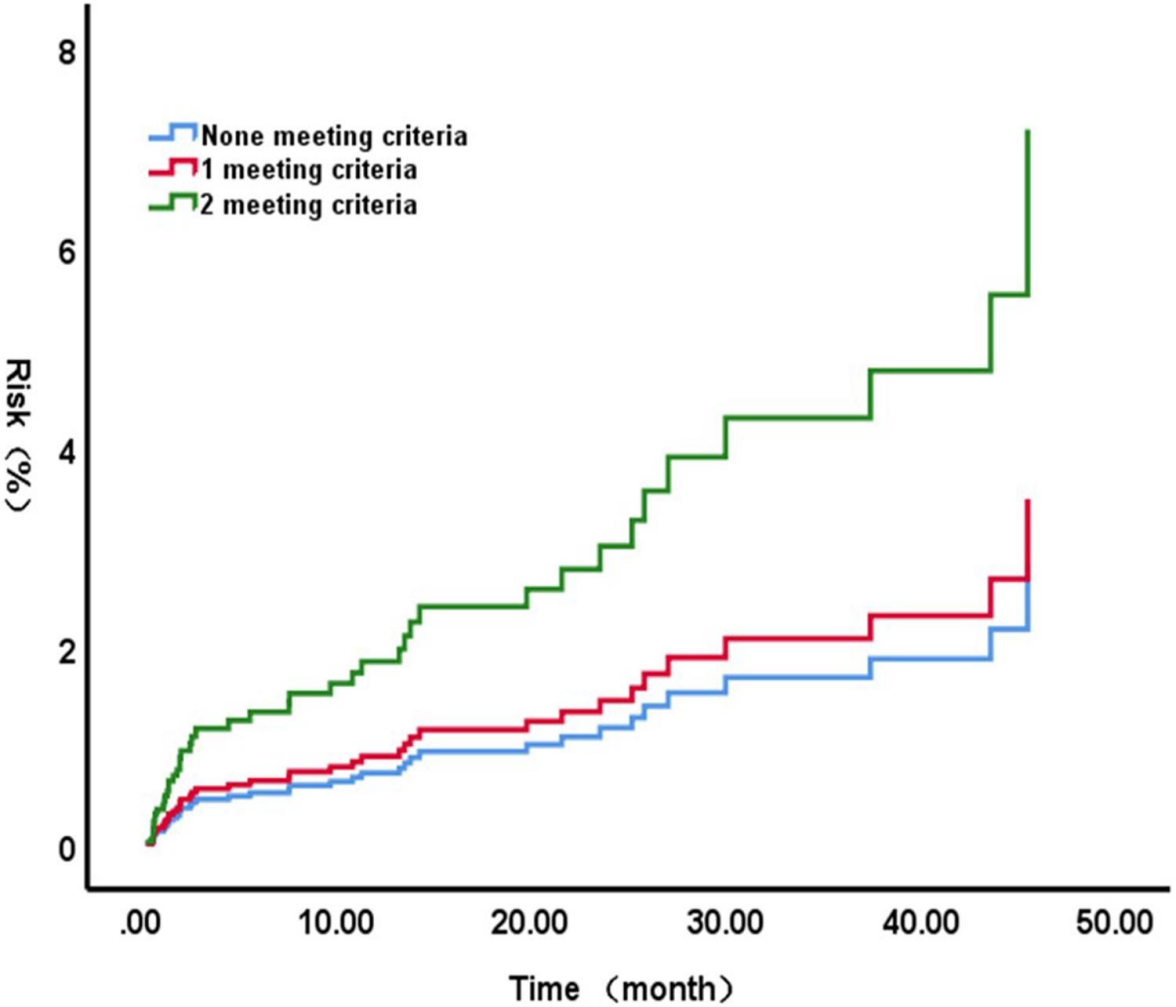
Kaplan-Meier survival curve for unplanned readmission rate by presence of markers.

Kaplan–Meier survival curves (Figure 3) were plotted based on the presence of 0, 1, or 2 risk factors (LVEDD > 65 mm and/or LVEF < 60%). The results further revealed a significantly increased risk of unplanned readmission after surgery in patients possessing both risk factors simultaneously.

## 4 Discussion

Current international guidelines recommend surgical intervention for patients with AR upon the onset of symptoms, a significant decline in LVEF (<55%), or severe left ventricular enlargement, such as an LVEDD exceeding 65 mm or an LVESD greater than 50 mm (3). However, the optimal timing for surgery and postoperative risks remain controversial for patients with LVEF between 55%–60% who already present with left ventricular dilation. This retrospective study demonstrated that even patients with only mildly impaired LVEF faced a significantly elevated risk of unplanned readmission after surgery (HR = 2.548, *P* = 0.018) when significant left ventricular dilation (LVEDD > 65 mm) was also present. Furthermore, patients with LVEF 55%–60% combined with LVEDD > 65 mm demonstrated substantially higher postoperative readmission rates compared to those presenting with only a single risk factor or no risk factors.

Our study demonstrated that LVEDD > 65 mm and LVEF < 60% serve as independent predictive factors for unplanned readmission following AR surgery. This finding suggests that the clinical risk stratification threshold for LVEF should be established at 60%, rather than the traditional 50% or 55%. AR patients with LVEF in the 50%–59% range (particularly 55%–60%) require the same level of attention as those with heart failure with reduced ejection fraction (HFrEF). In aortic valve disease, LVEF measured by transthoracic echocardiography is routinely employed to assess left ventricular systolic dysfunction and guide intervention, as its deterioration correlates with adverse short-term and long-term prognosis (10, 11). In fact, Left ventricular ejection fraction significantly underestimates the extent of systolic dysfunction in the left ventricular myocardium, which is a frequent finding among patients with AR. Multiple studies emphasize that LVEF < 50% lacks sensitivity for identifying patients with subclinical left ventricular systolic dysfunction who may be at higher risk of short-term adverse events and thus could benefit from early intervention (5, 12). During the early stages of chronic AR, the left ventricle maintains its ejection fraction through eccentric hypertrophy and dilation. Thus, left ventricular size may serve as a more sensitive indicator of early ventricular dysfunction than LVEF itself (2, 13). This study found that as LVEF decreased, left ventricular dimensions and volumes significantly increased, which aligns with the classic cardiac remodeling process (14). Even when the LVEF remains above 55%, myocardial fibrosis and extracellular matrix remodeling are already underway. This phenomenon termed “functional-structural decoupling” (15, 16), indicates that the impairment of myocardial contractile reserve precedes the decline in LVEF (17). Although surgery corrects valvular regurgitation, myocardial structural and functional recovery requires extended time, with some patients experiencing irreversible damage. Inadequate postoperative left ventricular reverse remodeling and insufficient cardiac functional reserve may lead to heart failure, arrhythmias, or valve-related complications, thereby increasing readmission risk (18, 19). LVEF demonstrated a nonlinear association with readmission risk (20, 21). Treatment disparities played a significant role, as patients with EF < 50% likely received more intensive and standardized guideline-directed medical therapy for heart failure, thereby reducing readmission rates. Conversely, patients with LVEF 55%–60% may not have been classified as having typical heart failure, resulting in insufficient therapeutic intensity. Additionally, this may relate to left ventricular diastolic dysfunction. While these patients maintained compensated systolic function, they exhibited elevated left ventricular filling pressures, which also explains why LVEDD > 65 mm emerged as another independent predictor. Our study found that gender, blood pressure, atrial fibrillation, renal function, atrial size, and right ventricular function showed no association with unplanned readmission after AR surgery. This further underscores that left ventricular function and structure (LVEF and left ventricular size) serve as crucial predictors for unplanned postoperative readmission (22–24).

Based on a risk stratification model incorporating two factors—LVEDD > 65 mm and LVEF < 60%—this study effectively differentiated the risk of unplanned readmission among AR patients. The presence of a single abnormal parameter indicated a trend toward increased risk. However, a substantial and statistically significant elevation in risk occurred only when severe structural remodeling (ventricular dilation) coexisted with functional impairment (reduced systolic function), reaching clinically meaningful levels. This indicates that when evaluating postoperative prognosis in AR patients, a comprehensive assessment of left ventricular functional and structural parameters should be performed rather than relying solely on any single isolated parameter. Current guidelines defining LVEF <55% as one surgical indication may overlook high-risk patients with LVEF between 55%–60% who already exhibit significant left ventricular dilation. For different risk stratifications, we recommend distinct clinical management approaches. Patients meeting both risk factors are classified as high-risk; clinicians should maintain vigilance through intensified pharmacotherapy, close follow-up monitoring, and patient education (25, 26). Additionally, regarding the non-significant risk observed in the “one risk factor” subgroup, larger sample sizes may be required to confirm or exclude its risk.

For high-risk patients (LVEF 55%–60% + LVEDD > 65 mm), we recommend structured echocardiographic surveillance at 3, 6, and 12 months postoperatively, then annually. Monitoring should focus on left ventricular reverse remodeling (LVRR). Global longitudinal strain (GLS) provides incremental prognostic value. Patients with inadequate LVRR by 6–12 months should be referred for multidisciplinary heart failure team evaluation.

This study also has several limitations. First, as a single-center, observational, retrospective study, it carries inherent limitations associated with this study design. Second, although the sample size was substantial, the limited number of readmission events may affect the stability of multivariate analysis. Third, our retrospective database did not systematically capture post-discharge guideline-directed medical therapy (GDMT) prescription data. Treatment disparities may play a significant role in our findings, as patients with EF < 50% likely received more intensive and standardized heart failure therapy, while those with LVEF 55%–60% may not have been recognized as requiring similar management intensity. This potential confounding effect warrants consideration when interpreting our results. Fourth, although our study was conducted at a single tertiary center with relatively homogeneous access to care, we were unable to fully account for other potential confounders such as socioeconomic status and post-discharge medication adherence. Furthermore, this study failed to incorporate more indicators reflecting left ventricular remodeling or systolic function (such as global longitudinal strain, GLS). Future research could explore this area using cardiac magnetic resonance or more sophisticated echocardiographic parameters. Prospective multicenter studies with systematic collection of GDMT data and longer follow-up periods are needed to validate our findings.

## 5 Conclusion

In summary, AR patients with LVEF between 55% and 60% and significant left ventricular enlargement (LVEDD > 65 mm) exhibit a significantly higher risk of unplanned readmission after surgery. Clinical practice should prioritize this population by implementing individualized assessment of surgical timing, while enhancing long-term postoperative follow-up and cardiac function management to improve patients' long-term prognosis.

## Data Availability

The original contributions presented in the study are included in the article/Supplementary Material, further inquiries can be directed to the corresponding authors.
